# GPI0363 inhibits the interaction of RNA polymerase with DNA in *Staphylococcus aureus*†

**DOI:** 10.1039/c9ra06844a

**Published:** 2019-11-21

**Authors:** Atmika Paudel, Suresh Panthee, Hiroshi Hamamoto, Kazuhisa Sekimizu

**Affiliations:** Teikyo University Institute of Medical Mycology 359 Otsuka, Hachioji Tokyo 192-0395 Japan sekimizu@main.teikyo-u.ac.jp; Genome Pharmaceuticals Institute 102 Next Building, 3-24-17 Hongo, Bunkyo-ku Tokyo 113-0033 Japan

## Abstract

We previously reported a therapeutically effective spiro-heterocyclic compound, GPI0363, that inhibits the transcription of *Staphylococcus aureus via* the primary sigma factor of RNA polymerase, SigA. Here, we demonstrated that GPI0363 shares no cross-resistance with the clinically used RNA polymerase inhibitors rifampicin and fidaxomicin. Furthermore, we found that GPI0363 bound to SigA of both GPI0363-susceptible and resistant strains, and inhibited the interaction of the RNA polymerase holoenzyme with DNA. In addition, the gene expression patterns following GPI0363 treatment were different from those following rifampicin treatment. These findings suggest that GPI0363 has a unique mechanism of action and can serve as a promising lead molecule to develop staphylococcal RNA polymerase inhibitors.

## Abstract 2

GPI0363 has a distinct mode of action *via* SigA and is active against bacteria resistant to clinically used RNAP inhibitors.

## Graphical abstract

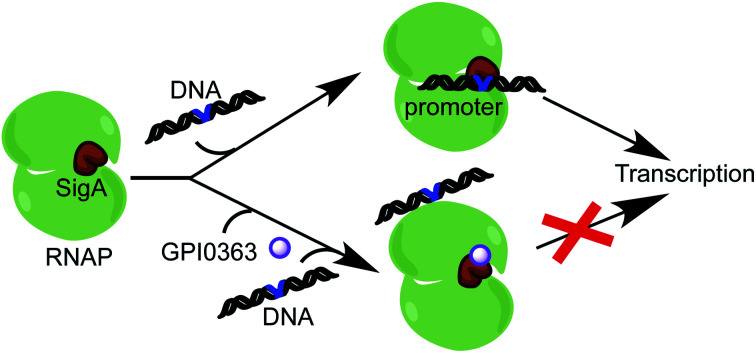

## Introduction

Bacterial RNA polymerase (RNAP), an enzyme involved in gene transcription and expression,^1^ is an attractive target for antimicrobials,^2^ mainly because it is conserved among prokaryotes. The eukaryotic transcription system, which comprises of RNA polymerases I, II, and III, typically differs from the prokaryotic transcription system; thus, selective toxicity to prokaryotes can be achieved by inhibiting RNAP. Two antimicrobial agents, rifampicin and fidaxomicin, are currently in clinical use; rifampicin acts by inhibiting the extension of short RNA products,^3^ and fidaxomicin inhibits the interaction of RNAP with promoter regions of DNA.^4^ Although RNAP inhibitors are thought to be broad-spectrum antimicrobial agents,^5,6^ narrow-spectrum RNAP inhibitors are also reported.^7,8^ Nonetheless, the identification of new RNAP inhibitors with novel mechanisms is critical for antimicrobial drug development to overcome the growing problem of drug-resistance.

**Fig. 1:**
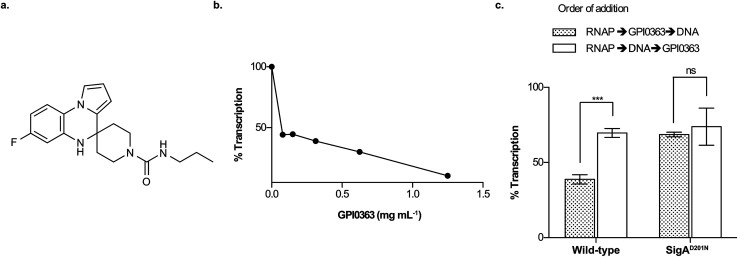
Transcription inhibition by GPI0363. (a) Structure of GPI0363. (b) Effect of GPI0363 on holoenzyme formation. *S. aureus* SigA was treated with GPI0363 (1.25, 0.62, 0.31, 0.15, 0.078 mg mL^−1^) before incubation with *E. coli* RNAP core enzyme followed by *in vitro* transcription. The transcripts were extracted, electrophoresed, and visualized by autoradiography. (c) Effect of GPI0363 on promoter-specific transcription before and after *S. aureus* RNAP (Sau RNAP) was incubated with DNA. Sau RNAPs from the wild-type and GPI0363-resistant mutant were treated with GPI0363 (0.3 mg mL^−1^) before or after incubation with template DNA followed by *in vitro* transcription. The transcripts were extracted, electrophoresed, and visualized by autoradiography. Data represent mean ± SD of three independent experiments and were analyzed by Student's *t*-test using Prism for Mac OS X, version 5.0d (GraphPad Software). Significant differences are indicated by asterisks (***p = 0.002). Band intensities were measured by ImageJ v1.47 by taking the band intensity without GPI0363 as 100%. Gel images are shown in ESI Fig. 3, 5 and 6.†

We previously reported a therapeutically effective spiro-heterocyclic antimicrobial, GPI0363 (Fig. 1a), identified by screening in a silkworm infection model.^8,9^ It inhibited transcription in *Staphylococcus aureus* by binding to the primary sigma factor, SigA, and a single point mutation (D201N) in SigA was responsible for resistance to GPI0363.^8^ However, the mechanism of how it inhibits transcription was not fully understood. Here, we elucidated that GPI0363 inhibited the interaction of RNAP holoenzyme with DNA and has a mechanism distinct from that of clinically used antibiotics.

## Results and discussion

### GPI0363 does not have cross-resistance with clinically used RNAP inhibitors

Given that GPI0363 targets RNA synthesis, we evaluated whether GPI0363 has cross-resistance with clinically used RNAP inhibitors. We generated spontaneous *S. aureus* strains resistant to rifampicin (R1–R5) and fidaxomicin (F1–F5) and determined their susceptibilities to GPI0363. We also determined the drug susceptibilities of the GPI0363-resistant strain G2-1, which was generated by phage transduction and has a D201N mutation in SigA.^8^ The strains resistant to rifampicin and fidaxomicin were susceptible to GPI0363 and *vice versa* (Table 1). We then analyzed the whole genome sequence of the rifampicin- and fidaxomicin-resistant strains and found that all the rifampicin-resistant strains had a single amino acid substitution, H418Y, in the RNAP β subunit RpoB (Table 1). The RpoB^H418Y^ mutation is reported in several rifampicin-resistant strains^10,11^ and is known to confer resistance by decreasing the binding affinity of rifampicin to the RNAP.^10^ Similarly, among five fidaxomicin-resistant strains, four (F1, F2, F4, and F5) had the same single amino acid substitution Q1061K in the RNAP β subunit, RpoB. This mutation is also reported in various fidaxomicin-resistant strains.^12,13^ The remaining one strain (F3) harbored an R80S substitution in the RNAP β′ subunit, RpoC (Table 1). RpoC R80 is one of six amino acid residues involved in hydrogen-bond formation with fidaxomicin and a mutation at this position is responsible for resistance to fidaxomicin.^4^ These results suggest that the mechanism of GPI0363 is distinct from those of rifampicin and fidaxomicin.

**Table 1:** Antimicrobial activities of GPI0363 and rifampicin or fidaxomicin against laboratory-generated drug-resistant strains^a^

Strain	Mutation	MIC (μg mL^−1^)		Strain	Mutation	MIC (μg mL^−1^)	
		GPI0363	Rifampicin			GPI0363	Fidaxomicin
R1	RpoB^H481Y^	4	>64	F1	RpoB^Q1061K^, PheT^A797V^, GlvC^V282I^	4	32
R2	RpoB^H481Y^	8	>64	F2	RpoB^Q1061K^	4	64
R3	RpoB^H481Y^	4	>64	F3	RpoC^R80S^	8	64
R4	RpoB^H481Y^	8	>64	F4	RpoB^Q1061K^, GlnP^N152Y^	2	64
R5	RpoB^H481Y^	4	>64	F5	RpoB^Q1061K^	4	64
G2-1	SigA^D201N^	32	0.004	G2-1	SigA^D201N^	32	2
WT		4	0.004	WT		4	2

a The MIC was determined by broth microdilution assay. The same results were obtained from two independent experiments.

### GPI0363 binds to mutant SigA from GPI0363-resistant strain

The absence of cross-resistance of GPI0363 with clinically used RNAP inhibitors led us to study the mechanism of GPI0363 in detail. In our previous study, we found that GPI0363 inhibits transcription in *S. aureus* by binding to the primary sigma factor SigA of the RNA polymerase, and a D201N mutation in SigA is responsible for resistance to GPI0363.^8^ We hypothesized that the mutant SigA might have little or no binding affinity with GPI0363, thus conferring resistance. Therefore, as a first step to study the mechanism of action, we checked the binding of GPI0363 to the wild-type and mutant SigA. GPI0363 bound to both SigAs with a 0.8 ratio of GPI0363 eluted from the beads with SigA^D201N^ to wild-type SigA (ESI Fig. 1†). The binding of GPI0363 to SigA from both the susceptible and resistant strains implied that resistance to GPI0363 could not be explained by the loss of binding capacity and D201 in SigA might not be involved in the binding to GPI0363. On this basis, we speculated that GPI0363 acts at latter steps – binding of SigA to the RNAP core enzyme or interaction of the RNAP holoenzyme with DNA.

### GPI0363 inhibits the interaction of RNA polymerase with DNA after binding to SigA

To examine the effect of GPI0363 in the different transcription steps, we first established an *in vitro* transcription assay using three different promoters of the *dra*, *pflB* (ESI Fig. 2†), and *fbaA*^8^ genes. We found that GPI0363 inhibited transcription from these promoters in a similar and dose-dependent manner. We selected the *fbaA* promoter for our subsequent experiments as it showed a clear promoter-specific band with less background among the three promoters. To test whether GPI0363 inhibits the binding of SigA to the RNAP core enzyme, we treated SigA with GPI0363, followed by the addition of the *Escherichia coli* RNAP core enzyme and performed *in vitro* transcription. We found that the transcription inhibition from this hybrid RNAP (Fig. 1b, and ESI Fig. 3†) was similar to that from the *S. aureus* RNAP (Sau RNAP) holoenzyme.^8^ Additionally, compared with the Sau RNAP holoenzyme,^8^ transcription inhibition from the *E. coli* RNAP holoenzyme required an approximately 10-fold higher concentration of GPI0363 (ESI Fig. 4†), suggesting that GPI0363 is specific for *S. aureus* SigA and might not affect the formation of the RNAP holoenzyme.

To test the ability of GPI0363 to inhibit the interaction of the RNAP holoenzyme with DNA, we added GPI0363 before and after incubation of the Sau RNAP holoenzyme with DNA and assessed the transcription *in vitro*. Stronger inhibition was observed when GPI0363 was added to the wild-type Sau RNAP before incubation with the DNA, whereas the transcription inhibition from mutant Sau RNAP was not affected by the different orders of addition (Fig. 1c, ESI Fig. 5 and 6†).

### GPI0363 did not intercalate into the double-stranded DNA

Next, to test whether GPI0363 interacts with DNA itself, we added salmon sperm DNA to the assay medium and evaluated the effect of external DNA on the minimum inhibitory concentration (MIC) of GPI0363. The MIC values of GPI0363 did not change in the presence or absence of external DNA (Table 2), while those of actinomycin D, a known DNA intercalator,^14^ increased in the presence of the external DNA, suggesting that GPI0363 does not intercalate with DNA.

**Table 2:** MIC values of GPI0363 in the presence and absence of external DNA. The MIC was determined against *S. aureus* MSSA1 by broth microdilution assay in the presence of salmon sperm DNA^a^

DNA concentration (μg mL^−1^)	MIC (μg mL^−1^)	
	GPI0363	Actinomycin D
0	4	0.125
7.8	4	1
15.6	4	1
31.3	4	2
62.5	4	2
125	4	>2
250	4	>2
500	4	>2
1000	4	>2

a The same results were obtained from two independent experiments.

**Fig. 2:**
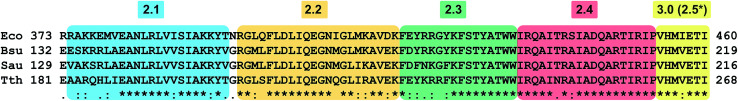
Sequence alignment of region 2 of SigA. The protein sequences were obtained from NCBI and aligned using Clustal Omega (https://www.ebi.ac.uk/Tools/msa/clustalo/). The regions were assigned according to Vassylyev *et al.*^18^ *Region 2.5 was later named region 3.0^15^. The accession number of proteins used for the analysis are: Eco (*E. coli*): AAC76103.1; Bsu (*B. subtilis*): CAB14450.2; Sau (*S. aureus*): BAF67736.1; Tth (*Thermus thermophilus*): BAD70355.1. The D201 position is indicated by an arrow.

Taken together, our findings suggest that GPI0363 inhibits the interaction between the RNAP holoenzyme and DNA by binding to SigA. To further gain an insight into the function of the mutation responsible for GPI0363 resistance, we aligned the *S. aureus* SigA with the SigAs from Gram-positive *Bacillus subtilis*, Gram-negative *E. coli*, and Gram-negative Deinococcus-Thermus *Thermus thermophilus*. The analysis revealed that D201 was in the universally conserved region 2.4 of SigA (Fig. 2). This region harbors allele-specific suppressors of promoter mutations in the −10 promoter region,^15^ thus involved in promoter recognition^16–18^ suggesting that D201N mutation affects the promoter-specific binding capacity of RNAP. A detailed structural analysis of the effect of this mutation and determination of the exact binding site of GPI0363 with SigA by co-crystallization is planned for a future study.

### GPI0363 treatment alters the *S. aureus* transcriptome differently than rifampicin

**Fig. 3:**
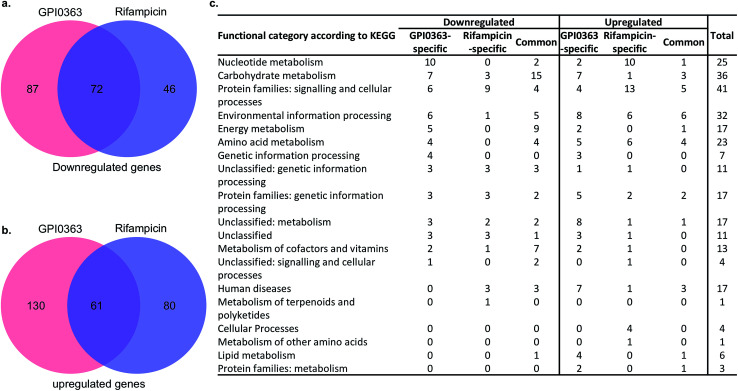
Differential expression of genes by RNA-seq analysis after treatment with GPI0363 and Rifampicin. (a) Venn diagram showing the number of genes commonly downregulated and (b) upregulated. (c) Functional categorization of significantly upregulated and downregulated genes after antibiotic treatment.

To further reveal the difference in the mechanisms of action between rifampicin and GPI0363, we performed RNA-seq analysis after treating *S. aureus* Newman with the MICs of the respective drugs. We compared the gene expression patterns on the basis of significant fold-expression changes [minimum fold difference: 2, false discovery rate (FDR) *p* value: <0.05] of GPI0363-treated and rifampicin-treated samples in comparison with the non-treated samples. Comparative analysis of downregulated (Fig. 3a) and upregulated (Fig. 3b) genes indicated that a large number of differentially expressed genes was distinct among the two drugs. We further performed a functional categorization of the upregulated and downregulated genes according to the KEGG pathway and found that the two drugs differentially affected the metabolic processes of *S. aureus* (Fig. 3c).

While genetic information processing was only affected by GPI0363, cellular processes, metabolism of terpenoids and polyketides, and metabolism of other amino acids were only affected by rifampicin. Nucleotide metabolism was mostly upregulated by rifampicin and mostly downregulated by GPI0363, and energy metabolism was more affected by GPI0363 than by rifampicin. Although changes in the gene expression patterns may have arisen as secondary effect, the results obtained here imply that the target genes of GPI0363 may be different from those of rifampicin.

## Experimental

### Chemicals and reagents

All the chemicals and reagents used in this study were of analytical grade. GPI0363 was obtained from the chemical library of the Drug Discovery Initiative at the University of Tokyo and its purity was confirmed by ultra-performance liquid chromatography-high resolution mass spectrometry (*m*/*z*: 343.1968 [M + H]^+^; calculated for C_19_H_24_N_4_O: 343.1934) (ESI Fig. 7†). Rifampicin (potency: 1029 μg mg^−1^), ammonium acetate, and HPLC grade acetonitrile were obtained from Fujifilm Wako Pure Chemical Industries, Ltd., Osaka, Japan. Fidaxomicin (purity: ≥95%) was obtained from Cayman Chemical (Ann Arbor, MI, USA). Salmon sperm DNA, actinomycin D (purity: ∼98%), and phenol : chloroform : isoamyl alcohol (125 : 24 : 1, v/v/v) were obtained from Sigma Aldrich, Tokyo, Japan. Tryptone, tryptic soy broth (TSB), yeast extract, and Mueller-Hinton broth (MHB) were obtained from Becton, Dickinson and Company (Franklin Lakes, NJ, USA). RNase inhibitor was obtained from Applied Biosystems (Beverly, MA, USA). ATP, CTP, GTP, UTP, and yeast tRNA were obtained from Ambion (Austin, TX, USA). [α-^32^P] UTP was obtained from PerkinElmer, Waltham, MA, USA. The *E. coli* RNAP holoenzyme and core enzyme were obtained from New England Biolabs (Ipswich, MA, USA), and TALON® magnetic beads were obtained from Takara Bio USA Inc. (Mountain View, CA, USA).

### Microorganisms and culture conditions

The bacterial strains used in this study are summarized in ESI Table 1.†*S. aureus* strains were grown in TSB and *E. coli* was grown in Luria Bertani medium. MHB supplemented with cations was used to determine antimicrobial susceptibility.

### Generation of spontaneous strains resistant to antibiotics

*S. aureus* RN4220 was cultured overnight on TSB at 37 °C with ambient air at 200 rpm. An aliquot (100 μL) of the overnight culture was then spread on TSB agar plates containing 4 and 8 μg mL^−1^ rifampicin, or 8 and 16 μg mL^−1^ fidaxomicin. The plates were incubated at 37 °C overnight, and single colonies were isolated.

### Binding of SigA with GPI0363

SigA from the wild-type and GPI0363-resistant *S. aureus* RN4220 strain was expressed in *E. coli* to obtain wild-type SigA and SigA ^D201N^, respectively as previously described.^8^ Binding of SigA with GPI0363 was evaluated according to the previously reported method.^8^ Briefly, TALON® magnetic beads pre-charged with cobalt were equilibrated with 50 mM Tris buffer, pH 7.5 followed by incubation with wild-type SigA or SigA ^D201N^ in the presence or absence of GPI0363. GPI0363 was pretreated with bovine serum albumin (0.5 mg mL^−1^) before incubating with SigA and beads. The resulting beads were washed with the same buffer, separated, and eluted with 50% acetonitrile + 0.1% trifluoroacetic acid, and analyzed by high-performance liquid chromatography (HPLC) with a TSKgel α-M size exclusion column (7.8 mm ID × 30 cm, 13 μm; TOSOH) and a mobile phase comprising 50% acetonitrile + 0.1% trifluoroacetic acid at a flow rate of 0.5 mL min^−1^.

### *In vitro* transcription assay

DNA templates were prepared with primers as indicated in ESI Table 2† using PrimeSTAR Max DNA Polymerase (TaKaRa, Japan). Purification of *S. aureus* RN4220 RNAP and *in vitro* transcription were performed as previously described.^8,19^ Briefly, 0.2 μL of the RNAP fraction was added to the reaction buffer (final volume 25 μL) containing 40 mM Tris-acetate (pH 7.9), 100 mM NaCl, 5 mM MgCl_2_, 0.2 mM DTT, 100 μg mL^−1^ bovine serum albumin, and 0.5 units of RNase inhibitor. GPI0363 was then added and the mixture was incubated for 5 min at 37 °C, before initiating the transcription by the addition of NTP mixture [0.25 mM each of ATP, CTP, GTP; 0.015 mM UTP, and 10 μCi of [α-^32^P] UTP with template DNA (a 406-bp fragment of the *fbaA* gene, a 528-bp fragment of the *pflB* gene, or a 486-bp fragment of the *dra* gene including the promoter regions)]. The reaction was allowed to proceed for 5 min at 37 °C and then stopped by the addition of 100 μL ice-cold stop solution (0.4 M ammonium acetate, 20 mM EDTA, 0.3% sodium dodecyl sulfate, 4 μg tRNA). Transcripts were extracted in phenol : chloroform : isoamyl alcohol (125 : 24 : 1, v/v/v); electrophoresed on 7 M urea, 6% polyacrylamide gels, and visualized by autoradiography using Typhoon FLA 9000 (GE Healthcare, Japan). The band intensities were quantified using ImageJ software (v1.47, NIH, USA).^20^ To examine the effect of GPI0363 in the interaction of RNAP and DNA, a reaction mixture containing the RNAP fraction was incubated at room temperature for 5 min with template DNA before the addition of GPI0363. For experiments with *E. coli* core RNAP, *S. aureus* SigA was treated with GPI0363 for 10 min at room temperature, followed by incubation with reaction mixture containing the *E. coli* RNAP core enzyme at room temperature for 10 min. The reaction was started with the addition of the mixture of NTPs and *fbaA* template DNA. For *in vitro* transcription from the *E. coli* RNAP holoenzyme, a 450-bp long DNA fragment from pBR322 was used as the template.

### *In vitro* susceptibility test of *S. aureus* to antimicrobials

*S. aureus* MSSA1 and RN4220 were grown on Luria Bertani (tryptone 10 g L^−1^, yeast extract 5 g L^−1^, NaCl 10 g L^−1^, pH 7.0) agar plates at 37 °C with ambient air at 200 rpm overnight. The MIC was determined by broth microdilution assay in cation-adjusted MHB according to previously described methods.^21,22^ For determination of the MIC in the presence of external DNA, we added salmon sperm DNA (deoxyribonucleic acid sodium salt from salmon testes, Sigma Aldrich) into the MHB medium, and actinomycin D (Sigma Aldrich) was used as a positive control.

### RNA extraction from *S. aureus*

The overnight culture of *S. aureus* Newman was diluted 100 fold and incubated at 37 °C with aeration until the OD_600_ was 1.0. The culture was treated with GPI0363 (MIC-4 μg mL^−1^) or rifampicin (MIC- 0.004 μg mL^−1^) for 30 min. To stabilize the RNA, 4 mL of RNAprotect Bacteria Reagent (Qiagen, Hilden, Germany) was added to 2 mL bacterial culture. Total RNA was extracted using the RNeasy® Protect Bacteria Mini Kit (Qiagen) according to manufacturer's protocol using QIAcube (Qiagen) with lysis in 10 mM Tris HCl, 1 mM EDTA buffer (pH 8.0) containing 1 mg mL^−1^ lysostaphin at 37 °C for 30 min.

### Library preparation for RNA-sequencing and analysis of data

Ribosomal RNA in the total RNA was depleted using MICROB*Express*™ Bacterial mRNA Enrichment Kit (Thermo Fisher Scientific, Carlsbad, CA, USA) according to the manufacturer's protocol and rRNA depletion was confirmed using an Agilent Bioanalyzer 2100 (Agilent Technologies, Santa Clara, CA, USA). Whole transcriptomic libraries for RNA sequencing were prepared according to the instructions for the Ion Total RNA-seq Kit v2 (Thermo Fisher Scientific). Briefly, rRNA-depleted RNA was fragmented using RNase III, followed by reverse transcription and amplification. The size distribution and yield of the amplified library was confirmed using the Bioanalyzer 2100. The libraries were then enriched in an Ion PI Chip v2 using the Ion Chef (Thermo Fisher Scientific), and the subsequent sequencing was performed in the Ion Proton System (Thermo Fisher Scientific). All data were analyzed using CLC Genomics Workbench ver. 12.0 (Qiagen Bioinformatics, Aarhus, Denmark). Reads were aligned to the Newman genome allowing a minimum length fraction of 0.9 and minimum similarity fraction of 0.9. Genes with a significant false discovery rate (*p* < 0.05) and minimum two-fold-expression were classified as having significantly different expression.

### Genomic DNA extraction and whole-genome sequence analysis

Genomic DNA of the strains was isolated from overnight cultures using a DNeasy Blood and Tissue kit (Qiagen) by QIAcube (Qiagen) according to the manufacturer's recommendations. The library was prepared using 100 ng of the genomic DNA quantified using a Qubit 3.0 fluorometer (Thermo Fisher Scientific) as described previously.^23,24^ Briefly, genomic DNA was fragmented using an Ion Shear™ Plus Reagent Kit (Thermo Fisher Scientific) and a 400 base-read barcoded library was prepared using the Ion Xpress™ Plus Fragment Library Kit (Thermo Fisher). E-Gel SizeSelect™ (Thermo Fisher Scientific) was used for the 400 base size selection, and the library was amplified by polymerase chain reaction. The quality, quantity, and size distribution of the libraries were determined using an Agilent Bioanalyzer 2100 (Agilent Technologies), and enriched using an Ion 318™ Chip v2 in Ion Chef (Thermo Fisher). Sequencing was performed in the Ion Personal Genome Machine (Thermo Fisher Scientific). The reads were then mapped to the *S. aureus* NCTC8325 genome, the parent strain of *S. aureus* RN4220, using CLC Genomics Workbench ver. 12.0 (Qiagen Bioinformatics) followed by the extraction of variants specific to the drug-resistant strains.

## Conclusions

Identifying antimicrobial agents effective against drug-resistant pathogens and understanding how these molecules exert their actions is critical towards combating the growing spread of multi drug-resistant pathogens. Recently, while screening of antimicrobial agents against methicillin-resistant *S. aureus*, we found that GPI0363 inhibits promoter-specific transcription by binding to the primary sigma factor of RNAP, SigA.^8^ The bacterial RNAP holoenzyme binds to the promoter region of template DNA and forms an open complex by separating the double helix of the template, followed by transcription initiation.^25^ In this manuscript, we investigated the mechanism of action of GPI0363 in detail and demonstrated that the anti-staphylococcal activity of GPI0363 is the result of transcription arrest caused by the inhibition of the interaction of the RNAP holoenzyme with the promoter region. Several antimicrobial agents, such as myxopyronin, corallopyronin, ripostatin, and fidaxomicin, target the switch region of RNA polymerase and interfere with the RNAP-DNA interaction.^4,26^ GPI0363 has a unique mode of action by binding to SigA. Moreover, the lack of cross-resistance of GPI0363 with rifampicin and fidaxomicin implies that its mechanism is distinct, which was further evidenced by the different transcriptomic profiles upon treatment with GPI0363 and rifampicin. Future studies should focus on elucidating the exact binding site and the sequence of events taking place in the *S. aureus* transcription machinery after GPI0363 treatment. In addition to being a promising lead molecule for the development of narrow-spectrum antimicrobial agents, GPI0363 can serve as a chemical biology tool for fundamental studies of SigA-dependent transcription.

## Conflicts of interest

There are no conflicts to declare.

## Supplementary Material

RA-009-C9RA06844A-s001

## References

[cit1] Murakami K. S., Darst S. A. (2003). Curr. Opin. Struct. Biol..

[cit2] Ho M. X., Hudson B. P., Das K., Arnold E., Ebright R. H. (2009). Curr. Opin. Struct. Biol..

[cit3] McClure W. R., Cech C. L. (1978). J. Biol. Chem..

[cit4] Lin W., Das K., Degen D., Mazumder A., Duchi D., Wang D., Ebright Y. W., Ebright R. Y., Sineva E., Gigliotti M., Srivastava A., Mandal S., Jiang Y., Liu Y., Yin R., Zhang Z., Eng E. T., Thomas D., Donadio S., Zhang H., Zhang C., Kapanidis A. N., Ebright R. H. (2018). Mol. Cell.

[cit5] Srivastava A., Talaue M., Liu S., Degen D., Ebright R. Y., Sineva E., Chakraborty A., Druzhinin S. Y., Chatterjee S., Mukhopadhyay J., Ebright Y. W., Zozula A., Shen J., Sengupta S., Niedfeldt R. R., Xin C., Kaneko T., Irschik H., Jansen R., Donadio S., Connell N., Ebright R. H. (2011). Curr. Opin. Microbiol..

[cit6] Maffioli S. I., Zhang Y., Degen D., Carzaniga T., Del Gatto G., Serina S., Monciardini P., Mazzetti C., Guglierame P., Candiani G., Chiriac A. I., Facchetti G., Kaltofen P., Sahl H.-G., Dehò G., Donadio S., Ebright R. H. (2017). Cell.

[cit7] Osmundson J., Montero-Diez C., Westblade L. F., Hochschild A., Darst S. A. (2012). Cell.

[cit8] Paudel A., Hamamoto H., Panthee S., Kaneko K., Matsunaga S., Kanai M., Suzuki Y., Sekimizu K. (2017). Front. Microbiol..

[cit9] Panthee S., Paudel A., Hamamoto H., Sekimizu K. (2017). Front. Microbiol..

[cit10] O'Neill A. J., Huovinen T., Fishwick C. W. G., Chopra I. (2006). Antimicrob. Agents Chemother..

[cit11] Wang C., Fang R., Zhou B., Tian X., Zhang X., Zheng X., Zhang S., Dong G., Cao J., Zhou T. (2019). BMC Microbiol..

[cit12] Babakhani F., Seddon J., Sears P. (2014). Antimicrob. Agents Chemother..

[cit13] Kurabachew M., Lu S. H. J., Krastel P., Schmitt E. K., Suresh B. L., Goh A., Knox J. E., Ma N. L., Jiricek J., Beer D., Cynamon M., Petersen F., Dartois V., Keller T., Dick T., Sambandamurthy V. K. (2008). J. Antimicrob. Chemother..

[cit14] Trask D. K., Muller M. T. (1988). Proc. Natl. Acad. Sci. U. S. A..

[cit15] Campbell E. A., Muzzin O., Chlenov M., Sun J. L., Olson C. A., Weinman O., Trester-Zedlitz M. L., Darst S. A. (2002). Mol. Cell.

[cit16] Paget M. S. (2015). Biomolecules.

[cit17] Chandrangsu P. and Helmann J. D., in eLS, John Wiley & Sons, Ltd, Chichester, 2014

[cit18] Vassylyev D. G., Sekine S.-i., Laptenko O., Lee J., Vassylyeva M. N., Borukhov S., Yokoyama S. (2002). Nature.

[cit19] Deora R., Misra T. K. (1996). J. Biol. Chem..

[cit20] Schneider C. A., Rasband W. S., Eliceiri K. W. (2012). Nat. Methods.

[cit21] Paudel A., Kaneko K., Watanabe A., Matsunaga S., Kanai M., Hamamoto H., Sekimizu K. (2013). J. Antibiot..

[cit22] Clinical and Laboratory Standards Institute , Methods for dilution antimicrobial susceptibility tests for bacteria that grow aerobically; approved standard—ninth edition (CLSI document M07–A9), Clinical and Laboratory Standards Institute, Wayne, PA, 2012

[cit23] Panthee S., Hamamoto H., Ishijima S. A., Paudel A., Sekimizu K. (2018). Genome Biol. Evol..

[cit24] Panthee S., Paudel A., Blom J., Hamamoto H., Sekimizu K. (2019). Front. Microbiol..

[cit25] Saecker R. M., Record M. T., deHaseth P. L. (2011). J. Mol. Biol..

[cit26] Mukhopadhyay J., Das K., Ismail S., Koppstein D., Jang M., Hudson B., Sarafianos S., Tuske S., Patel J., Jansen R., Irschik H., Arnold E., Ebright R. H. (2008). Cell.

