# Supplementary material for: GPI0363 inhibits the interaction of RNA polymerase with DNA in Staphylococcus aureus
Source: RSC Adv. 2019 Nov 21;9(65):37889–94. doi: 10.1039/c9ra06844a (PMC9075815; doi:10.1039/c9ra06844a)
Supplement: RA-009-C9RA06844A-s001 [file RA-009-C9RA06844A-s001.pdf]

Electronic Supplementary Material (ESI) for RSC Advances.
This journal is © The Royal Society of Chemistry 2019

Supplementary Information

GPI0363 inhibits the interaction of RNA polymerase with DNA in *Staphylococcus aureus*

Atmika Paudel[1,#], Suresh Panthee[1,#], Hiroshi Hamamoto[1], Kazuhisa Sekimizu[1,2*]

[1] Teikyo University Institute of Medical Mycology, 359 Otsuka, Hachioji, Tokyo 192-0395, Japan.

[2] Genome Pharmaceuticals Institute, 102 Next Building, 3-24-17 Hongo, Bunkyo-ku, Tokyo 113-0033, Japan

[#] These authors contributed equally to this work.

* Corresponding author: sekimizu@main.teikyo-u.ac.jp

Supplementary Table 1: Bacterial strains used in this study

Strain	Description	Source
Staphylococcus aureus RN4220	Restriction-deficient mutant derived from strain NCTC 8325.	Paudel et al., 2012[1]
S. aureus Newman	Methicillin-susceptible *S. aureus*	Baba et al., 2008[2]
S. aureus MSSA1	Methicillin-susceptible *S. aureus*, clinical isolate	Paudel et al., 2012[1]
F1-F5	Fidaxomicin-resistant spontaneous mutant derived from *S. aureus* RN4220	This study
R1-R5	Rifampicin-resistant spontaneous mutants derived from *S. aureus* RN4220	This study
G2-1, G2-2, G2-3, G2-20	GPI0363-resistant SigA^{D201N} mutants derived from *S. aureus* RN4220	Paudel et al., 2017[3]
Escherichia coli BL21(DE3)/pLysS	Cells for protein expression	Agilent Technologies

Supplementary Table 2: Primers used in this study

Purpose	Primer name	Primer sequence
Amplification of *pflB* promoter	*pflB*_Fwd	GGCAAGATATTGAAGGT
	*pflB*_Rev	TTGCTTCTGTTGGTCCTG
Amplification of *dra* promoter	*dra*_Fwd	TGTGGCGGTATCTGTAGGT
	*dra*_Rev	TGCTTTCGTTGCAGTTGT
Amplification of pBr322 promoter region	PBR_Fw	TTTCACCAGCGTTTCTGGGT
	PBR_rev	AGCCTATGCCTACAGCATCC

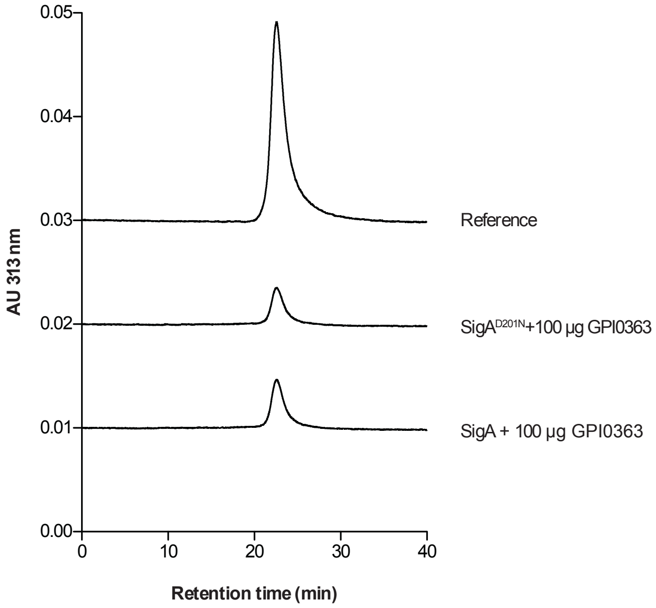

Supplementary Figure 1: Binding of GPI0363 to wild-type SigA and SigA ^{D201N}. Magnetic beads with His-tagged wild-type SigA or SigA ^{D201N} were incubated with GPI0363 pretreated with BSA; the bound fraction was washed, eluted, and analyzed by HPLC.

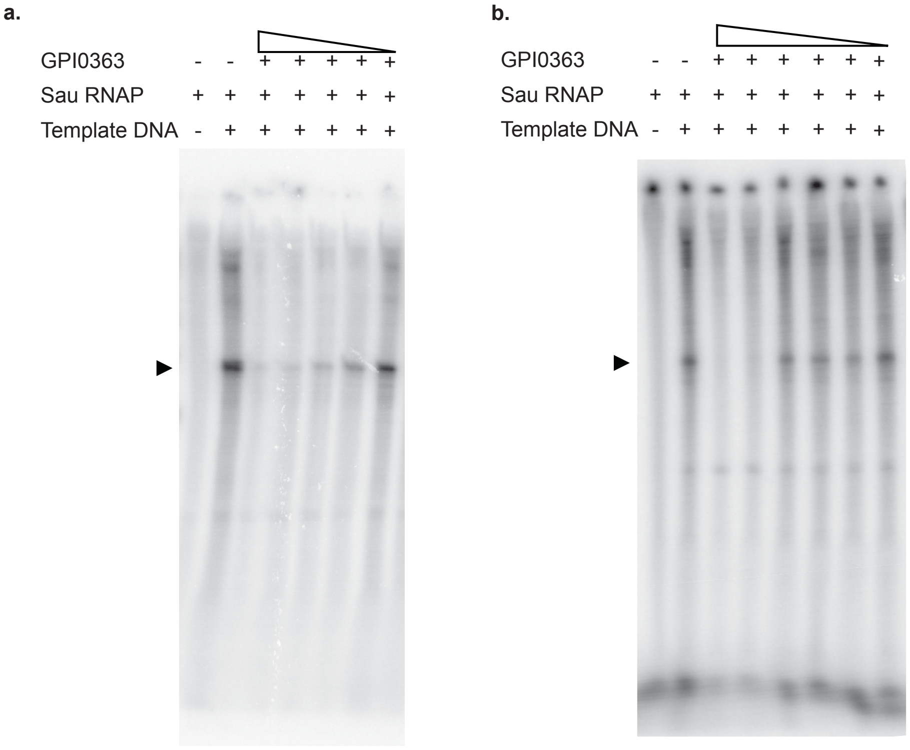

Supplementary Figure 2: ***In vitro*** **transcription inhibition in wild-type RNAP at different promoters. a.** In the presence of decreasing concentrations of GPI0363 (5, 2.5, 1.25, 0.62, 0.31 mg/mL) from *dra* promoter. **b.** In the presence of decreasing concentrations of GPI0363 (5, 1.25, 0.3, 0.07, 0.019, 0.0048 mg/mL) from *pflB* promoter. Promoter-specific transcripts are indicated by arrowheads.

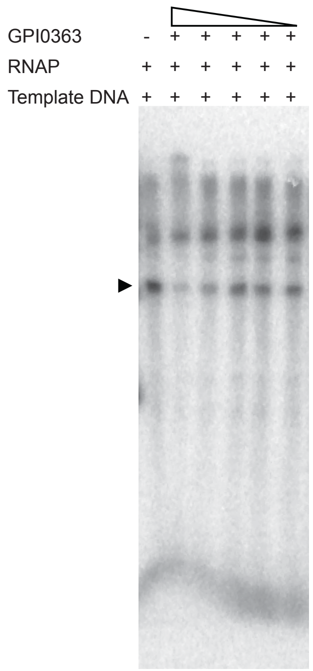

Supplementary Figure 3 (related to Figure 1b): *In vitro* transcription inhibition after treatment of wild-type SigA with GPI0363 before formation of the RNAP holoenzyme. Histidine-tagged recombinant SigA was purified from the wild-type. SigA was treated with GPI0363 (1.25, 0.62, 0.31, 0.15, 0.078 mg/mL) followed by reconstitution with *E. coli* RNA polymerase core enzyme and *in vitro* transcription was performed. Transcript was extracted and electrophoresed on 6% urea-polyacrylamide gel. Promoter-specific transcript is indicated by an arrowhead.

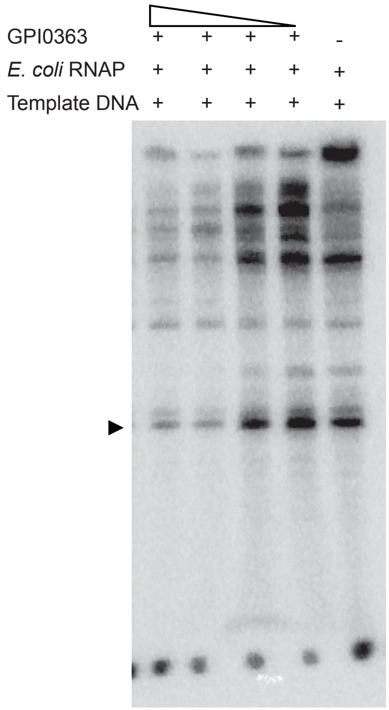

Supplementary Figure 4: Effect of GPI0363 in the *in vitro* transcription from *E.coli* RNAP. *In vitro* transcription was performed in the presence of decreasing concentrations of GPI0363 (5, 2.5, 1.0, 0.5, 0.25 mg/mL) from pBR322 promoter. Promoter-specific transcript is indicated by an arrowhead.

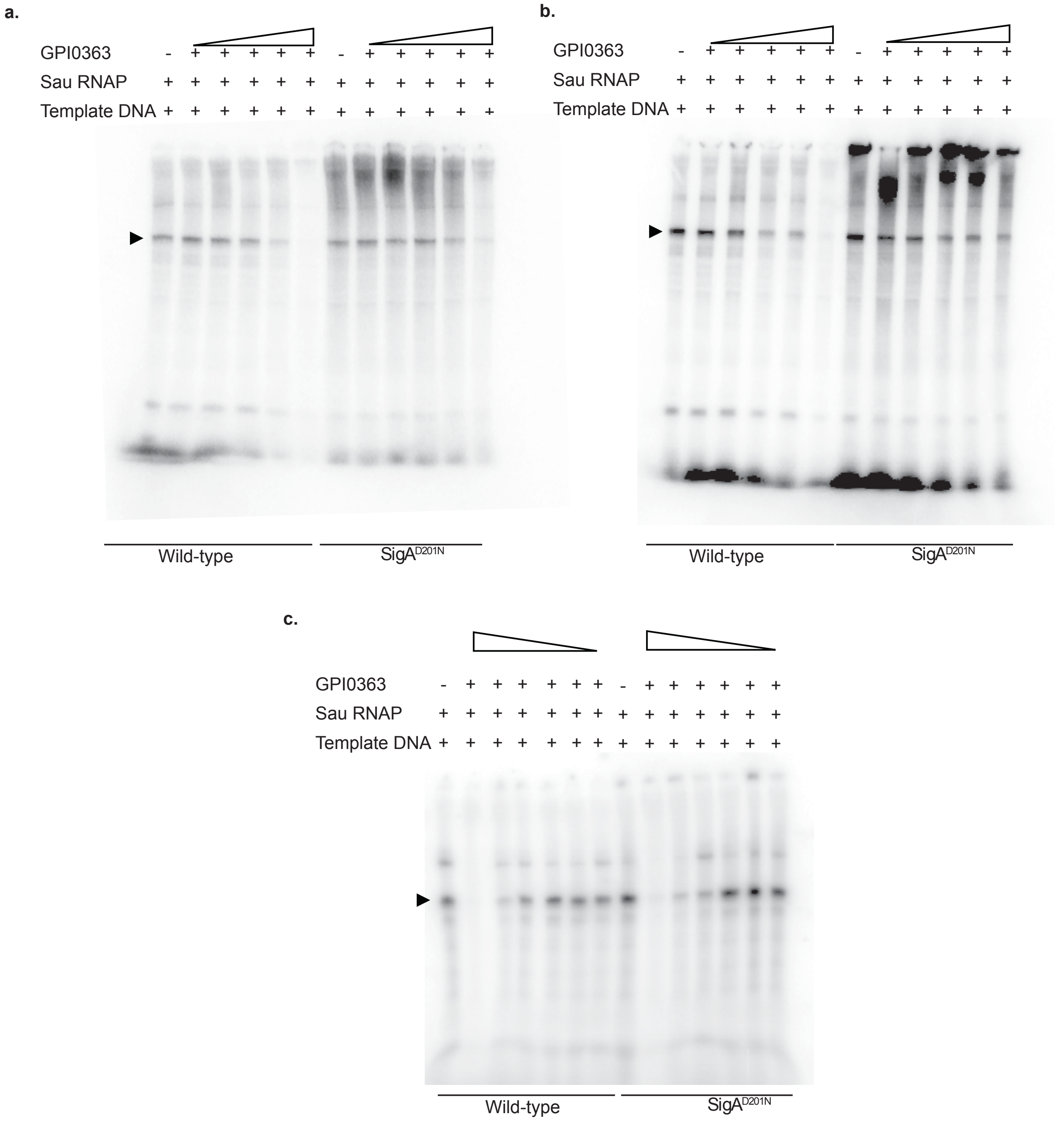

Supplementary Figure 5 (related to Figure 1c): *In vitro* transcription assay from wild-type and GPI0363-resistant RNAP when RNAP holoenzyme was treated with GPI0363 before interaction with DNA. **a.** and **b.** In the presence of increasing concentrations of GPI0363 (0.01, 0.05, 0.1, 0.3, 0.6 mg/mL). **c.** In the presence of decreasing concentrations of GPI0363 (5, 1.25, 0.31, 0.15, 0.016, 0.008 mg/mL). Promoter-specific transcripts are indicated by arrowheads.

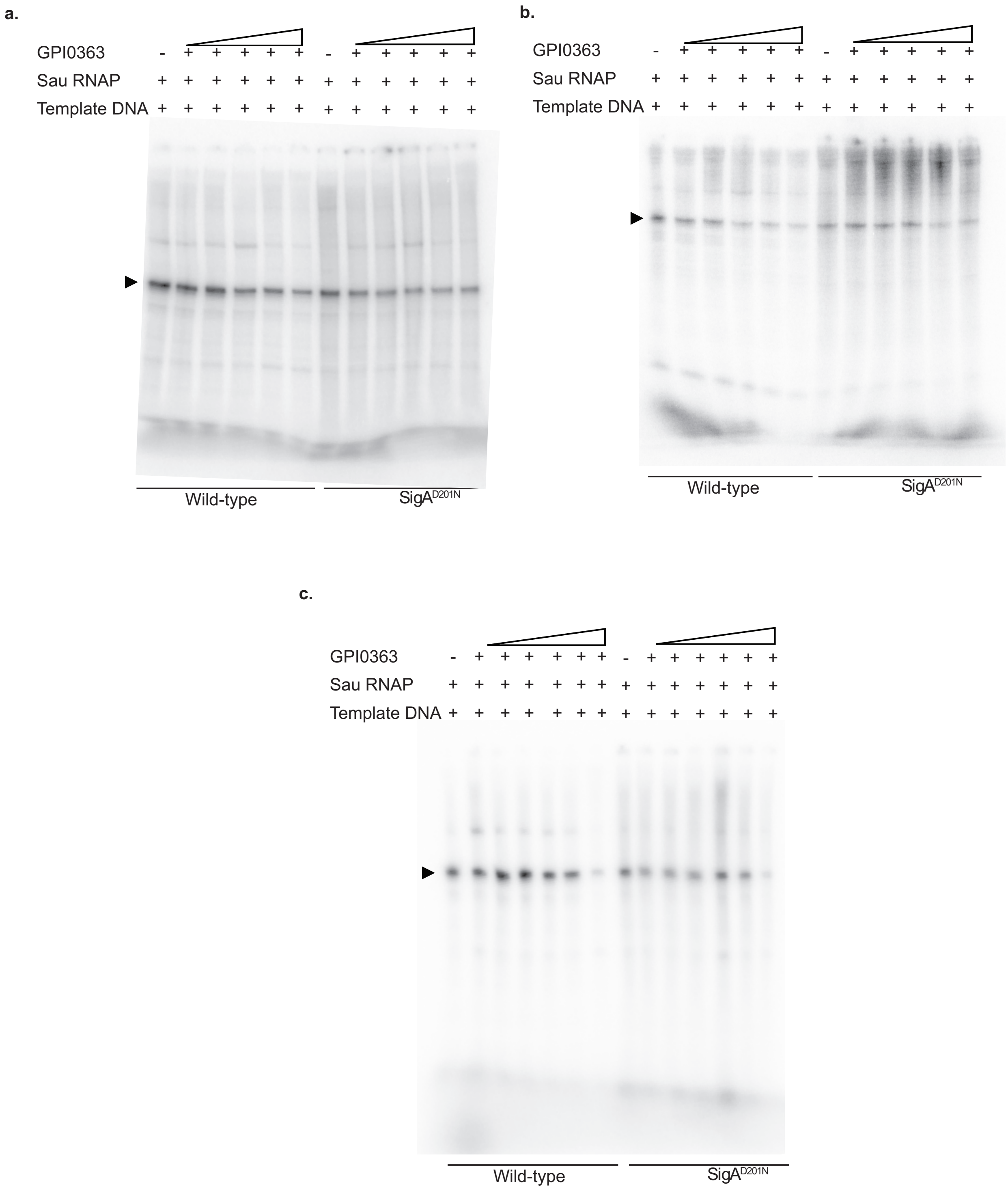

Supplementary Figure 6 (related to Figure 1c): *In vitro* transcription assay from wild-type and GPI0363-resistant RNAP when RNAP holoenzyme interacted with DNA before treatment with GPI0363. **a.** and **b.** In the presence of increasing concentrations of GPI0363 (0.01, 0.05, 0.1, 0.3, 0.6 mg/mL). **c.** In the presence of increasing concentrations of GPI0363 (0.01, 0.05, 0.1, 0.3, 2.0 mg/mL). Promoter-specific transcripts are indicated by arrowheads.

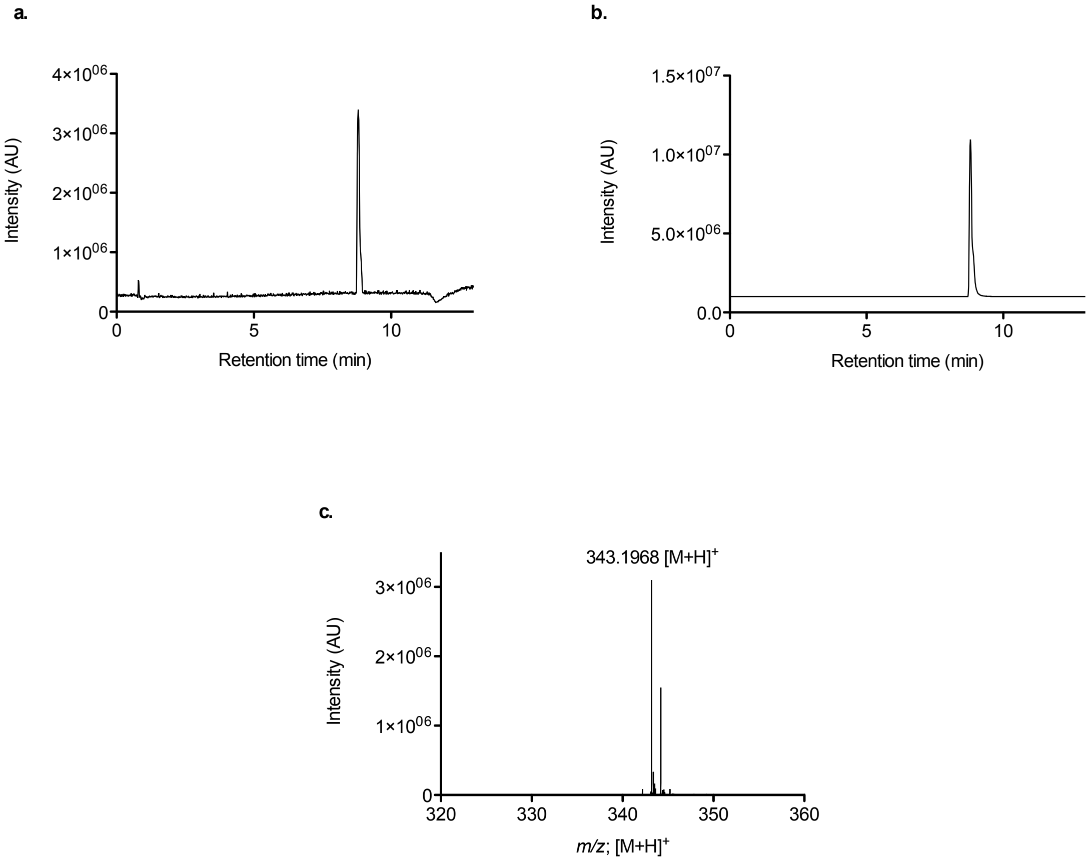

Supplementary Figure 7: UPLC/MS analysis of GPI0363. a. Total Ion Chromatogram **b.** Extracted Ion Chromatogram for 343.189 with the range of 0.05 Da. **c.** mass spectra of the peak eluted at 8.9 minutes. Mass analysis was performed in positive mode.

Supplementary References

1. A. Paudel, H. Hamamoto, Y. Kobayashi, S. Yokoshima, T. Fukuyama and K. Sekimizu, *J. Antibiot.*, 2012, **65**, 53-57.
2. T. Baba, T. Bae, O. Schneewind, F. Takeuchi and K. Hiramatsu, *J. Bacteriol.*, 2008, **190**, 300-310.
3. A. Paudel, H. Hamamoto, S. Panthee, K. Kaneko, S. Matsunaga, M. Kanai, Y. Suzuki and K. Sekimizu, *Front. Microbiol.*, 2017, **8**, 712.
